# Antioxidant Capacity of Free and Peptide Tryptophan Residues Determined by the ORAC (Oxygen Radical Absorbance Capacity) Assay Is Modulated by Radical-Radical Reactions and Oxidation Products

**DOI:** 10.3390/foods12234360

**Published:** 2023-12-02

**Authors:** Juan David Figueroa, Noreima Barroso-Torres, Marcela Morales, Bárbara Herrera, Mario Aranda, Eva Dorta, Camilo López-Alarcón

**Affiliations:** 1 Departamento de Química Física, Escuela de Química, Facultad de Química y de Farmacia, Pontificia Universidad Católica de Chile, Santiago 7820436, Chile; jdfigueroa1@uc.cl (J.D.F.); dmmorales2@uc.cl (M.M.); bherrera@uc.cl (B.H.); 2 Departamento de Producción Vegetal, Instituto Canario de Investigaciones Agrarias (ICIA), 38270 San Cristóbal de la Laguna, Spain; noreimabt97@gmail.com; 3 Escuela de Farmacia, Facultad de Química y de Farmacia, Pontificia Universidad Católica de Chile, Santiago 7820436, Chile; mario.aranda@uc.cl

**Keywords:** ORAC, tryptophan oxidation, bioactive peptides, antioxidant capacity, peroxyl/alky radicals, AAPH

## Abstract

The ORAC (Oxygen Radical Absorbance Capacity) assay is commonly employed for determining the antioxidant capacity of bioactive peptides. To gain insights into the meaning of this index for peptides containing a single Trp, we studied the consumption of this residue and fluorescein (FLH, the probe of ORAC method), induced by radicals generated by AAPH (2,2′-Azo-bis(2-amidinopropane) dihydrochloride) thermolysis. ORAC values were rationalized from kinetics and computational calculations of bond dissociation energies (BDE) of the N-H bond (indole ring of Trp). Free Trp, di- and tri- peptides, and three larger peptides were studied. Solutions containing 70 nM FLH, 1–5 μM free Trp or peptides, and 10 mM AAPH were incubated at 37 °C in phosphate buffer. Kinetic studies showed that FLH minimally affected Trp consumption. However, a clear protection of FLH, characterized by pseudo-lag times, was evidenced, reflecting radical-radical reactions and FLH repairing. Peptides showed similar ORAC values (~1.9–2.8 Trolox equivalents), while BDE varied between 91.9 and 103.5 kcal. These results, added to the protection of FLH observed after total consumption of Trp, indicate a lack of discrimination of the assay for the chemical structure of peptides and the contribution of oxidation products to the index.

## 1. Introduction

In the last years, an increased interest from researches and the industry has emerged in finding new natural antioxidants with high activity and low toxicity. For this purpose, the antioxidant capacity (AC) of isolated natural products, as well as their complex matrices, specifically bioactive peptides obtained from foods, has been determined [1,2,3]. Considering the complexity of the systems, in vitro assays originally developed to quantify the AC of enriched polyphenolic samples (i.e., fruit extracts and beverages) [4] have been applied to bioactive peptides and their mixtures [5,6]. From these studies, the use of bioactive peptides with the capacity to neutralize the reactive species commonly generated in foods has been suggested as antioxidant strategy with advantages over those based on synthetic derivatives. The in vitro AC of bioactive peptides is usually determined using methods to measure the bleaching of stable radicals (2,2-Diphenyl-1-picrylhydrazyl, DPPH, and 2,2′-azino-bis(3-ethylbenzothiazoline-6-sulfonic acid) radical anion, ABTS); the capacity of peptides to reduce ferric ions (ferric reducing antioxidant power, FRAP); and to evaluate the ability of peptides to inhibit the oxidation of a particular probe (oxygen absorbance capacity, ORAC) [7,8,9,10,11,12,13,14,15,16,17]. Studies regarding the structure-function of antioxidant peptides have been reported, giving insights into the influence of the amino acids and their sequence in a particular peptide on the AC [1,2,5,6,18]. Currently, there is a consensus that the in vitro AC not necessarily reflect the positive effects on human health associated with the intake or in vivo generation of bioactive peptides [1,2,5,6,18]. Nonetheless, it is accepted that the AC of these species provides valuable information in terms of the potential use of bioactive peptides, for example, as preservatives for foods and foodstuffs. Due to the ORAC assay being positioned as an important tool to determine the AC of peptides, we have analyzed the meaning of this index for bioactive peptides containing a single tryptophan (Trp), a highly susceptible residue to oxidation. As discussed below, in line with previous reports [19,20,21], our analysis, based on kinetics of Trp consumption and ORAC determinations, evidenced that the chemistry behind the ORAC assay is not simple, involving the formation of alkoxyl radicals, as well as reactions mediated by secondary radicals. ORAC values of free Trp, di- and tri- peptides showed similar values to hexa-, hepta-, and octa- peptides, suggesting that the ORAC assay does not discriminate amongst the types of Trp residues. The obtained results, which were rationalized by computational studies, suggest that the ORAC values of peptides with a single Trp residue do not obey simple mechanisms of reactions with the participation of secondary radicals and oxidation products.

## 2. Materials and Methods

### 2.1. Chemicals

2,2′-Azo-bis(2-amidinopropane) dihydrochloride (AAPH), fluorescein (FLH), Trolox (6-hydroxy-2,5,8-tetramethyl chroman-2-carboxylic acid), Trp, N-acetyl-Trp (N-Ac-Trp), dipeptides (Gly-Trp, Trp-Gly, Trp-Phe, and His-Trp), and tripeptides (Gly-Trp-Gly, and Gly-His-Gly) were purchased from Sigma–Aldrich (St. Louis, MO, USA). Asn-Ile-Thr-Gly-Trp-Leu, Ser-Val-Trp-Ile-Gly-Gly-Ser-Ile, and Glu-Val-Trp-Lys-Ser-Asp-Glu were supplied by GL Biochem (Shanghai) Ltd. (Shanghai, China). All compounds were employed as received.

### 2.2. Solutions

Stock solutions of free Trp, di- and tri- peptides, and Trolox were prepared daily in phosphate buffer (75 mM, pH 7.4) at concentrations between 10 and 100 μM. Stock solutions of Asn-Ile-Thr-Gly-Trp-Leu, Ser-Val-Trp-Ile-Gly-Gly-Ser-Ile, and Glu-Val-Trp-Lys-Ser-Asp-Glu were prepared by dissolving 7 mg of peptides in 100 μL of 1 M NaOH. These solutions were 5000-fold diluted in 75 mM sodium phosphate buffer pH 7.4. Stock solutions of AAPH (0.6 M) and FLH (500 nM) were prepared daily in phosphate buffer.

### 2.3. Consumption of Free and Peptides Trp Residues

Consumption of free and peptide Trp residues, induced by AAPH-derived radicals, was assessed by following the changes in the intrinsic fluorescence of Trp. Reaction mixtures (3 mL) containing free Trp or peptides (0.5 or 1 μM final concentrations) and 10 mM AAPH were incubated in 75 mM phosphate buffer (pH 7.4 at 37 °C) in a quartz cuvette placed in the thermostatized holder of a Perkin Elmer LS-55 spectrofluorimeter (Beaconsfield, UK).

Fluorescence intensity at 360 nm (excitation wavelength = 280 nm) of peptides was employed to follow the consumption induced by AAPH-derived radicals. The addition of AAPH to samples induced a fast decrease in the fluorescence intensity at 360 nm (excitation wavelength = 280 nm), which is attributed to a filter effect [22]. During the incubation of free Trp or peptides with AAPH, a progressive decrease in intensity was observed without changes in the shape of the fluorescence spectra [22]. Obtained kinetic data were adjusted to non-linear functions, from which the first derivate was determined. Values of the first derivate at zero reaction time were employed to calculate initial consumption rates (R_0_), expressed as μM/min.

In some experiments, the consumption of fluorescence (emission and excitation wavelength = 360 and 280 nm, respectively) of free Trp and peptides (1 μM final concentrations), induced by AAPH-derived radicals, was followed in the presence of 70 nM FLH. For these experiments, to solutions (3 mL) containing Trp or peptides, 81 μL of FLH (2.6 μM) were added, and the volume adjusted to 2950 μL with phosphate buffer (75 mM, pH 7.4). Solutions were kept at 37 °C, and 50 μL of 0.6 M AAPH were added.

### 2.4. ORAC Methodology

200 μL of solutions containing 70 nM FLH, 10 mM AAPH, prepared in 75 mM phosphate buffer (pH 7.4), in the absence and presence of free Trp or peptides, were incubated at 37 °C in wells of a Multi-Mode microplate reader (SynergyTM HT; Biotek Instruments, Winooski, VT, USA). The fluorescence of FLH was determined at 515 nm (excitation wavelength = 493 nm). Values of normalized fluorescence (F/F_0_) were plotted as a function of the incubation time with integration of the area under the curve (AUC) performed up to a time such that F/F_0_ reached 0.2. Plots and integration data were obtained using Graphpad Prism version 10 or MicroCal Origin^®^ 7.0 software. ORAC values were calculated, according to:

$$ORAC=\frac{{\left(AUC\right)}_{additive}-{\left(AUC\right)}_{0}}{{\left(AUC\right)}_{Trolox}{-(AUC)}_{0}}\times \frac{\left[Trolox\right]}{\left[Additive\right]}$$

where AUC = area under the curve in the absence or presence of the additive (free Trp or peptides), integrated between time zero and that corresponding to 80% (F/F_0_ = 0.2) of the probe consumption; (AUC)_additive_ = area under the curve in the presence of free Trp, N-Ac-Trp, or peptides; (AUC)_0_ = area under the curve for the control (FLH plus AAPH solution); and (AUC)_Trolox_ = area under the curve for Trolox; [Trolox] = Trolox molar concentration; [additive] = Molar concentration of free Trp, N-Ac-Trp, or peptides. All experiments were carried out in triplicate at least three independent days.

### 2.5. Computational Analysis

The structures of free Trp and peptides were optimized at the B3LYP/6-311g level using the opt = ModRedundant keyword (to keep the terminal N-H distances frozen to ensure the zwitterionic structures). All calculations were performed in Gaussian 16 Revision B.01. Figures of peptides were made using Gaussview 6.0.16 for MacOS [23].

The bond dissociation energy (BDE) was calculated using the following equation:

$$BDE=\left({BE}_{{Free\;Trp\;or\;Peptide}^{•}}+{BE}_{H^{•}}\right)-{BE}_{Free\;Trp\;or\;Peptide}$$

where BE = bond energy, with BE_Free Trp or Peptide^•^_ and BE_H^•^_ defined as the bond energy resulted from homolytic cleavage.

### 2.6. Data and Statistical Analysis

**Figure 1:**
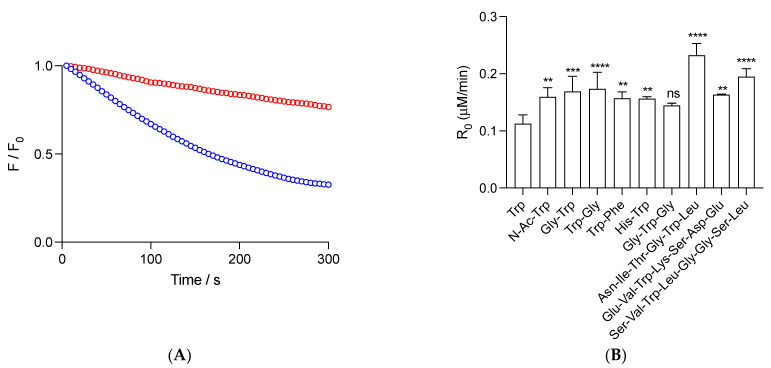
Radicals derived from AAPH thermolysis induce the consumption of free Trp and peptides. (**A**): Kinetic profiles of 1 (blue) and 5 (red) μM Gly-Trp. (**B**): Initial consumption rates (R_0_) of 1 μM Trp and peptides. Solutions of free Trp and peptides, prepared in phosphate buffer (75 mM, pH 7.4), were incubated in the presence of 10 mM AAPH at 37 °C. Consumption of the Trp and peptides was assessed following their fluorescence emission at 360 nm (exc. = 280 nm). Data represent the mean of at least three independent experiments. In panel B, standard deviations are indicated. Except for Gly-Trp-Gly, all data showed statistical differences with free Trp at *p* < 0.05 (**), *p* < 0.001 (***), and *p* < 0.0001 (****). ns = non-significant statistical difference.

Reported data correspond to the mean of at least three independent experiments, each carried out in triplicate. The analysis was developed by employing Graphpad Prisma 7.0a software. Statistical analysis of data presented in Figure 1 was carried out using a one-way ANOVA test with Tukey post-hoc test, with *p* < 0.05 taken as statistically significant.

## 3. Results and Discussion

The ORAC assay is based on the changes elicited by antioxidants in the area under the curve of the kinetic profiles of the consumption of FLH (followed by fluorescence) triggered by AAPH-derived radicals [24]. In spite of the experimental simplicity of the methodology, standardized by Ou and coworkers in 2001 [24], the complexity of the chemistry of the assay makes it difficult to rationalize the results. Part of this complexity is explained by the production of multiple species during the thermolysis of AAPH [25]. AAPH first decomposes to alkyl radicals (R^•^, reaction (1)), which react with O_2_ to generate peroxyl radicals (ROO^•^, reaction (2)). In the presence of low concentrations (or absence) of targets, the latter radicals self-react to produce a tetroxide intermediate (ROOOOR), whose decomposition generates alkoxyl radicals (RO^•^) and O_2_ (reactions (3) and (4)) [25]. These reactions affect the ORAC index since low concentrations of FLH (usually 70 nM) are employed In the assay, implying that the processes are mostly mediated by RO^•^ instead of ROO^•^ [19], as inferred from the reactivity of RO^•^ compared to ROO^•^ (E^0^′ = 1.6 and 1.0 V, respectively) [26]. Additionally, the use of low concentrations of FLH implies that this probe is easily protected by samples, giving kinetic profiles characterized by the presence of lag times, meaning that obtained values mainly represent the stoichiometry of the reactions [19,20,25].

(1) $$AAPH\to 2R^{•}+N_{2}$$

(2) $$R^{•}+O_{2}\to {ROO}^{•}$$

(3) $${2ROO}^{•}\to ROOOOR$$

(4) $$ROOOOR\to {2RO}^{•}+O_{2}$$

When the AC of free and peptides Trp residues is evaluated by the ORAC methodology and added to the complexity of the assay, the chemistry of the oxidation of Trp should be considered [27,28]. As depicted in reaction (5), the oxidation of Trp, mediated by AAPH radicals, involves a first step where a hydrogen atom is transferred from the indole ring of Trp to ROO^•^ (or RO^•^). In this reaction, a secondary radical (tryptophanyl radical, Trp^•^) is produced, which reacts with O_2_ (k = 1–5 × 10^5^ M^−1^s^−1^ [29]) to generate Trp peroxyl radicals (Trp-OO^•^). Trp-OO^•^ mediate chain reactions with the formation of hydroperoxides (reactions (6) and (7)), and other non-radical products such as carbonyl groups and alcohols (reaction (8)) [27,28]. It has been reported that the mechanisms of oxidation of free, peptides, and protein Trp residues, triggered by radicals derived from AAPH (6 mM, 45 °C), depend on the initial concentrations of the targets [22]. At low concentrations, oxidation is mainly initiated by RO^•^ with a low extent of chain reactions. In contrast, at high concentrations, the reactions would be mostly mediated by ROO^•^, with the process significantly influenced by chain reactions mediated by Trp-OO^•^ [22]. Based on kinetic studies, it was suggested that at low Trp concentrations, the extent of termination reactions involving Trp^•^ is affected by the inclusion of Trp in peptides. In short peptides, dismutation of Trp^•^ (reaction (9)) would be hindered, affecting the stoichiometry of the process [22]. Contrarily, this process (reaction (9)) would be favored in free Trp, decreasing (two-fold) the initial reaction rate [22]. Furthermore, dimerization of Trp^•^ to form Trp-Trp (reaction (10)), a recognized species participating in the crosslinking of peptides and proteins, should also be considered as a termination process [30,31].

(5) $${ROO}^{•}/{RO}^{•}+Trp\to {Trp}^{•}+ROOH/ROH$$

(6) $${Trp}^{•}+O_{2}\to {TrpOO}^{•}$$

(7) $${TrpOO}^{•}+Trp\to TrpOOH+{Trp}^{•}$$

(8) $${ROO}^{•}/{RO}^{•}+{Trp}^{•}\to non\;radical\;products$$

(9) $${Trp}^{•}+{Trp}^{•}\to {Trp}_{ox}+Trp$$

(10) $${Trp}^{•}+{Trp}^{•}\to TrpTrp$$

We carried out kinetics to follow the consumption of free and peptide Trp residues under the experimental conditions of the ORAC assay. Studied peptides were selected due to the presence in their structure of a single Trp, together with low reactive amino acids towards peroxyl radicals, as deduced from competitive reactions of amino acids and 5-amino salicylic acid towards AAPH-derived peroxyl radicals [32]. Figure 1A depicts representative results of the consumption of Gly-Trp mediated by radicals derived from the thermolysis of 10 mM AAPH (37 °C). As presented, a constant decrease in fluorescence over the time frame of the experiments was observed, reaching after 300 s, ~33 and 77% of the Initial Intensity for 1 and 5 μM Gly-Trp, respectively. From this type of kinetics, we determined the initial consumption rate (R_0_) of this peptide, as well as free Trp, N-Ac-Trp, Gly-Trp, Trp-Gly, Trp-Phe, His-Trp, Gly-Trp-Gly, Asn-Ile-Thr-Gly-Trp-Leu, Ser-Val-Trp-Ile-Gly-Gly-Ser-Ile, and Glu-Val-Trp-Lys-Ser-Asp-Glu (Figure 1B). At 1 μM, peptides showed R_0_ values higher (not statically different for Gly-Trp-Gly) than free Trp (R_0_ = 0.11 ± 0.01 μM/min), which, in agreement with previous reports [22], could be explained by changes in the stoichiometry of termination reactions. Dipeptides, Gly-Trp-Gly, and N-Ac-Trp showed R_0_ between 1.3- and 1.5-fold higher values than free Trp, suggesting that, in addition to the dismutation of Trp^•^ (which should be reflected in R_0_ values two times higher than free Trp), other processes could be altering R_0_. Interestingly, similar behavior was observed for Asn-Ile-Thr-Gly-Trp-Leu, Ser-Val-Trp-Ile-Gly-Gly-Ser-Ile, and Glu-Val-Trp-Lys-Ser-Asp-Glu, with R_0_ values of 0.23 ± 0.02, 0.16 ± 0.01, and 0.20 ± 0.01 μM/min, respectively.

(11) $$FLH+{RO}^{•}\to {FL}^{•}+ROH$$

(12) $$AH+{FL}^{•}⇆A^{•}+FLH$$

As mentioned above, the use of a low concentration of FLH in the ORAC assay implies that this probe is easily protected by antioxidants, generating lag times in the kinetic profiles. In fact, the protection of FLH given by Trolox, a hydro-soluble vitamin E analog, widely used as an antioxidant standard, is characterized by the presence of lag times in the kinetic profiles [19,20,24]. This behavior has been explained by Bisby and coworkers [33], by repairing of FLH where reactions of its secondary radical (FL^•^) and antioxidants (AH) (reactions (11) and (12)) are present.

**Figure 2:**
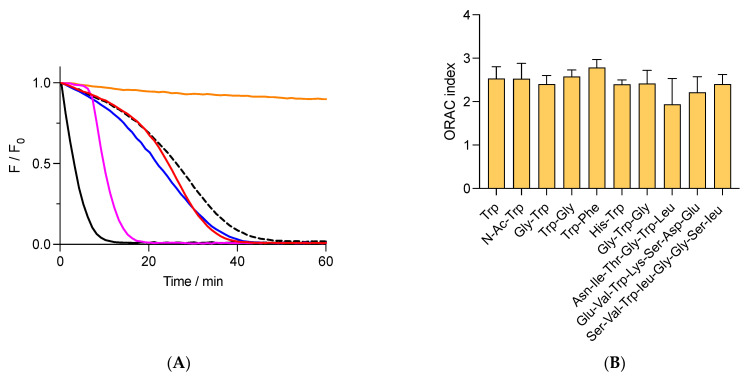
Free and peptide Trp residues inhibit the consumption of fluorescein (FLH) mediated by AAPH-derived radicals. FLH (70 nM) was incubated in phosphate buffer (75 mM, pH 7.4) at 37 °C in the presence of 10 mM AAPH and 1 μM of additives. (**A**) Kinetic profiles of FLH consumption in the absence (black) or presence of free Trp (- - -), Gly-Trp (blue), and Trp-Gly (red). Orange line = incubation of FLH without AAPH and additives. Pink line = protection of FLH afforded by 1 μM Trolox. (**B**) ORAC values of free Trp and peptides. Data represent the mean of at least three independent experiments, each carried out in triplicate. Fluorescence of FLH was followed at 515 nm (exc. = 493 nm).

When AH has low reduction potentials (E′^0^ for the process A^•^, H^+^/AH), reaction (12) is moved to FLH, favoring its repairing and the formation of lag times in the kinetic profiles [33]. The E′^0^ value of Trolox (0.480 V, [26]) explains the lag times generated by this compound, which contrasts with the absence of lag times observed when FLH is protected by tyrosine (E′^0^ = 0.930 V) [33,34]. Considering that free Trp has a higher redox potential than tyrosine (E′^0^ = 1.015 V for free Trp, [34]), its addition to solutions of FLH and AAPH should induce a protection of FLH associated with kinetic profiles without lag times. Nonetheless, the kinetics registered in the presence of free Trp and peptides showed that FLH was protected by kinetics with the presence of pseudo lag times, characterized by a slow decrease in fluorescence during the first minutes of incubations (Figure 2A). The area under the curve of these kinetics did not show differences between free Trp and peptides, implying that the AC determined by the ORAC assay is similar for these species. Results depicted in Figure 2B show that the ORAC assay does not discriminate if Trp is included in a particular peptide, with an average of the ORAC value of 2.4 ± 0.2 Trolox equivalents for all studied compounds. Similar experiments but developed with Gly-His-Gly showed very low protection of FLH with an ORAC of 0.08 ± 0.01 Trolox equivalents, confirming that Trp residues are the main species responsible for the antioxidant capacity. Remarkably, the ORAC values of Asn-Ile-Thr-Gly-Trp-Leu, Ser-Val-Trp-Ile-Gly-Gly-Ser-Ile, and Glu-Val-Trp-Lys-Ser-Asp-Glu were 1.9 ± 0.6, 2.4 ± 0.2, and 2.2 ± 0.3, respectively, which were also similar to those for free Trp, di- and tri- peptides (Figure 2B). This confirms that the ORAC assay, at least for the peptides herein studied (di-, tri-, hexa-, hepta-, and octa- peptides), is not controlled by the structure of the peptides.

**Figure 3:**
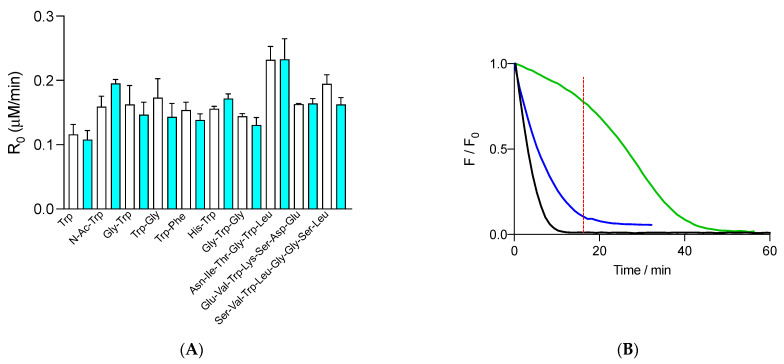
(**A**) Initial consumption rates (R_0_) of 1 μM free Trp and peptides determined in the presence of 10 mM AAPH and in the absence (white columns) and presence (cyan columns) of 70 nM FLH. Free Trp and peptides were employed at 1 μM initial concentration. (**B**) Kinetic profiles of FLH (green) and free Trp (blue) consumption in solutions containing 70 nM FLH, 1 μM Trp, and 10 mM AAPH incubated in phosphate buffer (75 mM, pH 7.4) at 37 °C. FLH and free Trp were followed by fluorescence at 515 and 360 nm, with 493 and 280 nm as excitation wavelengths, respectively. The black line represents the control of FLH consumption (FLH incubated with AAPH in the absence of free Trp). Data represent the mean of at least three independent experiments, each carried out in triplicate. Dashed red line shows the time where Trp was almost totally consumed.

These results indicate that the protection of FLH by free and peptide Trp residues does not obey simple reaction mechanisms, with the pseudo lag times probably resulting from the participation of secondary radicals generated in the system (Trp^•^ and FL^•^). To study the consumption of free Trp and peptides when incubated under the same experimental conditions as the ORAC assay, we followed the fluorescence of Trp residues during the time frame of their incubations in the presence of 70 nM FLH and 10 mM AAPH. For free Trp and peptides, the shape of the kinetics was not altered by the presence of 70 nM FLH. Only slight changes (without statistical significance) were observed in R_0_ values (Figure 3A). These results suggest that the oxidation of Trp residues evolved almost independently of the presence of FLH, with the production of Trp^•^ (reaction (5)) and FL^•^ (reaction (11)) mostly initiated by RO^•^. The observed protection of FLH mediated by Trp could be explained by reactions between Trp and FL^•^, as depicted in reaction (13), where a low fraction of Trp would participate in the repairing of FLH. In addition, based on the redox potential of Trp^•^, either calculated by computational methods or experimentally determined (E′^0^ = 0.85 and 1.03 V, respectively [35]), radical-radical reactions between Trp^•^ and FL^•^, leading to FLH, cannot be discarded (reaction (14)), a reaction competing with reaction (13) and self-reactions of FL^•^ (reaction (15)).

(13) $$Trp+{FL}^{•}⇆{Trp}^{•}+FLH$$

(14) $${Trp}^{•}+{FL}^{•}\to {Trp}_{ox}+FLH$$

(15) $${FL}^{•}+{FL}^{•}\to FLFL$$

Interestingly, when the consumption of free and peptides Trp residues, registered in the presence of FLH, was compared with the kinetic profiles of FLH consumption, protection of FLH after the total consumption of Trp was observed. As presented in Figure 3B, the fluorescence of free Trp (1 μM) was totally abolished after 16 min, a time when the fluorescence of FLH was ~77% of the initial intensity. After 16 min of incubation, the fluorescence of FLH followed a kinetic profile with a slower decrease than control experiments, implying that oxidation products of Trp can, at least partially, protect FLH.

**Figure 4:**
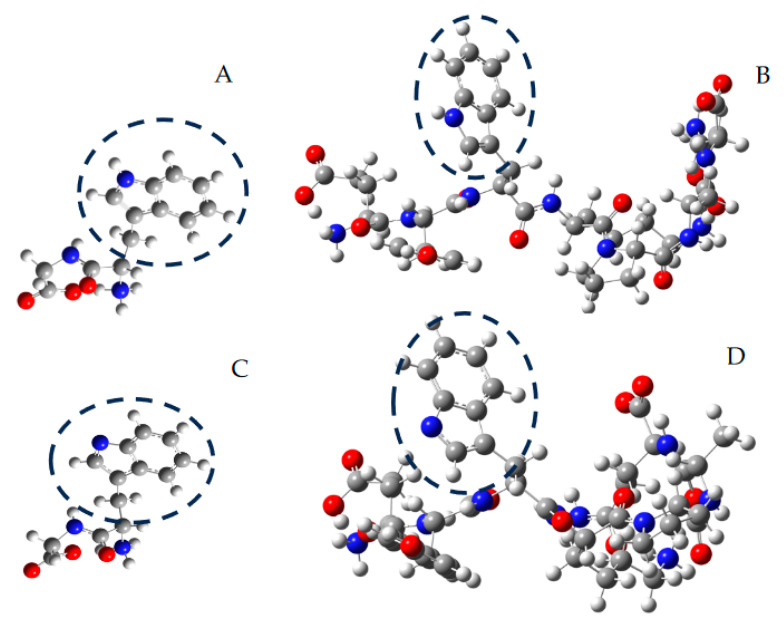
Representative structures, optimized by in silico studies at the B3LYP/6-311g level, of Trp-Gly (**A**), Ser-Val-Trp-Ile-Gly-Gly-Ser-Ile (**B**), and their secondary radicals ((**C**,**D**), respectively). Carbon, nitrogen, and oxygen atoms are represented as grey, blue, and red spheres, respectively. For illustrative purposes, the indole ring of Trp residues is circumscribed by dashed circles.

Aimed at gaining insights into how the inclusion of Trp in peptides could or could not modulate its susceptibility to oxidation, we developed computational analyses to determine the homolytic bond dissociation energies (BDE) of N-H in Trp residues. As presented in Table 1, BDE values ranging from 91.9 to 103.5 kcal were determined for free Trp and peptides. For Ser-Val-Trp-Ile-Gly-Gly-Ser-Ile, a BDE value of 101.4 kcal was calculated, which is similar to Glu-Val-Trp-Lys-Ser-Ala-Glu (103.5 kcal). It should be noted that the latter value represents a preliminary determination. This was obtained by using a coarse integration grid due to convergence problems caused by the low intramolecular interactions and thus, the higher degrees of freedom that Glu-Val-Trp-Lys-Ser-Ala-Glu presents in contrast to Ser-Val-Trp-Ile-Gly-Gly-Ser-Ile. Obtained BDE values showed that the lability of the N-H bonds varied by ~10 kcal, a difference not clearly evidenced in the ORAC values (it should be noted that the errors of the experimental ORAC measurements were higher than 10%). In silico calculations (Figure 4 shows optimized structures for Trp-Gly and Ser-Val-Trp-Ile-Gly-Gly-Ser-Ile, and their respective radicals) would suggest that peptides with different structures (di-, tri-, hexa-, hepta-, and octa- peptides) have a similar behavior towards AAPH-derived radicals, which could be associated with the similar values of the ORAC assay. Interestingly, a BDE value of 77.6 kcal was calculated for FLH, suggesting that its one-electron oxidation is favored in comparison with Trp and peptides. Therefore, the formation of FL^•^ and the occurrence of reactions (13)–(15) are favored.

## 4. Conclusions

Analysis of kinetic profiles of the consumption of Trp and fluorescein, induced by radicals derived from AAPH thermolysis, along with the redox potential of Trp and the BDE values of the N-H bond in the indole ring of Trp, denotes that the ORAC index for free Trp and peptides containing a single Trp involves a complex set of reactions. Among these reactions, those including radical-radical reactions, the repair of fluorescein, and the participation of oxidation products, influence antioxidant activity. Therefore, the ORAC values of these kinds of peptides do not reflect simple reaction mechanisms, implying that the antioxidant capacity evaluated by this assay should be carefully interpreted.

## Figures and Tables

**Table 1:** Initial consumption rates (R_0_, μM/min), ORAC (Trolox equivalents), and BDE values (kcal) of free Trp, peptides, and FLH. R_0_ and ORAC values represent the mean ± standard deviation of at least three independent experiments, each carried out in triplicate. * R_0_ was obtained at 1 μM for free Trp and peptides and 70 nM for FLH.

Free Trp or Peptide	R_0_ * (μM/min)	ORAC	BDE (kcal)
Trp	0.11 ± 0.02	2.5 ± 0.3	102.5
N-Ac-Trp	0.16 ± 0.02	2.5 ± 0.4	97.5
Gly-Trp	0.17 ± 0.03	2.4 ± 0.2	91.9
Trp-Gly	0.17 ± 0.03	2.6 ± 0.2	98.4
Trp-Phe	0.16 ± 0.01	2.8 ± 0.2	97.0
His-Trp	0.16 ± 0.01	2.4 ± 0.1	100.3
Gly-Trp-Gly	0.14 ± 0.01	2.4 ± 0.3	93.3
Asn-Ile-Thr-Gly-Trp-Leu	0.23 ± 0.02	1.9 ± 0.6	---
Glu-Val-Trp-Lys-Ser-Asp-Glu	0.16 ± 0.01	2.2 ± 0.4	103.5
Ser-Val-Trp-Ile-Gly-Gly-Ser-Ile	0.20 ± 0.01	2.4 ± 0.2	101.4
Fluorescein (FLH)	0.014	---	77.6

## Data Availability

All the data presented in this study are available in this article.
